# Involvement of RAGE and Oxidative Stress in Inflammatory and Infectious Skin Diseases

**DOI:** 10.3390/antiox10010082

**Published:** 2021-01-09

**Authors:** Fabrizio Guarneri, Paolo Custurone, Valeria Papaianni, Sebastiano Gangemi

**Affiliations:** 1 Department of Clinical and Experimental Medicine, Dermatology, University of Messina, Via Consolare Valeria—Gazzi, 98125 Messina, Italy; paolo.custurone@gmail.com (P.C.); valeriapapaianni82@hotmail.it (V.P.); 2 School and Operative Unit of Allergy and Clinical Immunology, Department of Clinical and Experimental Medicine, University of Messina, Via Consolare Valeria—Gazzi, 98125 Messina, Italy; sebastiano.gangemi@unime.it

**Keywords:** RAGE, sRAGE, skin, alarmins, cytokines, inflammation, autoimmunity, AGE, AOPP

## Abstract

The surface receptor for advanced glycosylation end-products (RAGE) and its soluble (sRAGE) and endogenous secretory (EN-RAGE) forms belong to the superfamily of toll-like receptors and play important roles in inflammation and autoimmunity, directly or through binding with advanced glycosylation end-products (AGE) and advanced oxidation protein products (AOPP). We reviewed the literature on the role of RAGE in skin diseases. Research in this field is still rather limited (28 articles) but suggests the involvement of RAGE and RAGE-related pathways in chronic inflammatory diseases (lupus, psoriasis, atopic dermatitis, and lichen planus), infectious diseases (leprosy, *Staphylococcus aureus*-induced skin lesions), alterations of the repairing processes in diabetic skin, systemic sclerosis, and ulcers. These data prompt further research in this field, which not only will be useful to better understand the pathogenetic mechanisms of diseases, but is also likely to have intriguing clinical implications. Indeed, when their role in the complex and multifactorial inflammatory balance will be adequately defined, RAGE and related molecules could be used as markers of disease severity and/or response to treatment. Moreover, future promising therapeutic perspectives could be topical administration of some of these molecules (e.g., sRAGE) to modulate local inflammatory response and/or the development of anti-RAGE antibodies for systemic treatment.

## 1. Introduction

Oxidation is a physiological and necessary part of the complex network of physical and chemical phenomena related to life. Oxidative reactions are involved, for example, in the biological chain of aerobic metabolism, in the biosynthesis of small and big molecules, in signal transduction between the outside and inside of cells as well as between the different cell compartments, and in the defense mechanisms used by immune cells [1,2]. However, quantitatively excessive and too long oxidation phenomena are linked to cellular suffering and destruction, which result, at the organism level, in several inflammatory, neoplastic, and degenerative diseases [3]. In this typically hormetic situation, where the beneficial or detrimental effect of reactive oxygen species (ROS) and electrophiles depends on their amount [4], it is not surprising that cells and living organisms have developed several enzymatic and non-enzymatic homeostatic mechanisms to adaptively maintain the delicate and dynamic oxidoreductive (redox) equilibrium within the limits of the so-called “eustress”, as opposed to the aforementioned detrimental imbalance, to which the term “oxidative stress” is commonly referred [5].

In normal conditions, ROS can be generated by the mitochondrial electron transport chain, proteins of the peroxisomal and endoplasmic reticulum, the Fenton reaction, as well as specific enzymes, like cyclooxygenases, nicotinamide adenine dinucleotide phosphate oxidases, lipoxygenases, and xanthine oxidases [6]. External factors, like smoking, eating/drinking habits or lifestyle, may also influence the production of ROS. Environmental variables, and the control of their effects on the redox status of cells, are particularly important in the case of skin, which is the outermost and largest organ of the human body and is structured to be an active interface structure, with multiple connections with the main homeostatic systems of the organism. The major environmental cause of ROS production in the skin is ultraviolet (UV) radiations, mainly derived from sun exposure. Many cutaneous cromophores may directly absorb UV and generate ROS: DNA and other nucleic acids, urocanic acid, tryptophan, tyrosine, NADH (reduced form of nicotinamide adenine dinucleotide), quinones, flavins, and porphyrins mostly absorb ultraviolet B (UVB), while trans-urocanic acid, melanin precursors, and riboflavin absorb ultraviolet A (UVA). Some authors also demonstrated that electromagnetic radiations with lower energy, i.e., visible and infrared light, may induce ROS formation in the skin [7]. Pollutants, like airborne particulate matter or ozone, may increase ROS and/or deplete cutaneous antioxidants [8]. To counterbalance oxidative stress, many antioxidants are available in the skin, including vitamins C and E, beta-carotene, ferritin, uric acid, coenzyme Q, glutathione, glutathione peroxidases, catalase, heme oxygenase, and superoxide dismutase [9].

However generated, excessive ROS may induce cell and organ suffering through multiple mechanisms, such as telomere shortening, DNA damage, mutations of mitochondrial DNA, microRNA (dys)regulation, alteration of matrix metalloproteinases and signal transduction pathways, modifications of the extracellular matrix, chronic inflammation (clinically evident or subclinical), protein oxidation, vascular alterations, and accumulation of advanced glycation end products (AGEs).

AGEs are biomolecules of various nature (proteins, lipids, nucleic acids) that are abnormally covalently bound by glucose or fructose, with the final effect of inhibition of their function. The term “glycation” is used to distinguish this process from glycosilation, a physiological enzyme-mediated binding of sugars that occurs at specific sites of target molecules to enable their functions [10]. Scientific interest in AGEs has grown rapidly in the past decades: published papers went from less than 10 per year in 1991 to about one thousand per year in the last seven years (2014–2020). The reason for this interest is their involvement in a wide and yet incompletely explored range of diseases, as well as biologic processes that are physiological but can be significantly influenced by extrinsic factors [11]. In the latter group, a process of dermatological interest is skin aging, which depends on time but can be enhanced by ROS production, UV exposure, pollutants, lifestyle, and/or personal habits. Among cutaneous molecules, glycated elastin is subject to abnormal aggregation and unusually interacts with lysozyme, glycated collagen resists to the proteolytic action of matrix metalloproteinases, and vimentin undergoes perinuclear accumulation, reducing the cellular contractile capacity; altogether, these modifications result in the tissue stiffening and reduced elasticity that are typically observed in aged skin [11]. Other than having direct detrimental effects, AGEs induce further alterations typical of skin aging through interaction with their receptor, RAGE (Receptor for Advanced Glycation End products) [12].

Discovered by Neeper et al. in 1992 [13], RAGE is a receptor that belongs to the superfamily of toll-like receptors and consists of two cytoplasmatic domains and a variable extracellular domain. Both domains are present in the surface receptor form, while the former domain is missing in the soluble form of the receptor (sRAGE) and the endogenous secretory form (EN-RAGE). In addition to AGEs, which are its canonical ligands, RAGE is also able to bind a number of other endogenous and exogenous molecules. Endogenous ligands belong to the group of DAMPs (Damage-Associated Molecular Patterns) or alarmins, molecules that are released by cells under stress conditions and after their death, are involved in the host’s defense immune system and act, through RAGE, as promoters of inflammation and cytokine production [14]. These alternative RAGE ligands include Advanced Oxidation Protein Products (AOPP) [15], markers of oxidative stress generated by the interaction of plasma proteins, mainly albumin, with chlorinated oxidants, like chloramines and hypochlorous acid [16]. This group of markers includes dityrosine, pentosidine, and carbonyl-containing protein products [17]. AOPP seem to play a role mostly in acute processes of response to inflammatory insults and are emerging as crucial factors in the development of a great deal of skin diseases with acute manifestations, such as systemic lupus erythematosus, vitiligo, psoriasis, and Behcet’s disease, even though other studies involving chronic and tumoral diseases (such as mastocytosis and melanoma) have shown an increase of their circulating levels in patients’ sera [18].

Other known alternative RAGE ligands are S100A12 (S100 calcium-binding protein A12, which is also known as “extracellular newly identified RAGE binding protein” or calgranulin C), High-Mobility Group Box 1 (HMGB1), phosphatidylserine on apoptotic cells, the complement protein C1q, DNA, and Gram negative bacterial lipopolysaccharide (LPS) [15]. Additionally, in other studies, binding to RAGE has been inferred, although without direct demonstration, for diesel particulate matter, oxidized low density lipoprotein, and extracellular heat shock protein 70 [15].

The membrane isoform of RAGE is implicated in the pathways of NFAT (nuclear factor of activated T-cells), NF-κB (nuclear factor kappa-light-chain-enhancer of activated B cells), STAT3 (signal transducer and activator of transcription 3), and CREB (cAMP response element-binding protein) transcription factors, which are involved in the autophagy and apoptosis processes. RAGE has been found in a large number of cell types, which include, among others, endothelial cells, neural cells, cardiac myocytes, mesangial cells, as well as cells of the immune system such as monocytes, macrophages, and T lymphocytes [19]. In the skin, RAGE is highly expressed, with variable patterns that depend on age and sun exposure [20]. In detail, in fetal skin, RAGE is located in the upper epidermis and in few endothelial cells within the dermis; in young skin, it is present in the superficial and middle epidermis and in the papillary dermis; in aged skin, it can be found in the middle and basal part of the epidermis and in the reticular dermis. Chronically sun-exposed skin shows patchy distribution of RAGE in the epidermis and increased expression of RAGE on cells of the papillary dermis. For which concerns the type of cells, RAGE was mainly expressed on fibroblasts, dendrocytes, and endothelial cells, while the number of RAGE-positive CD45 lymphocytes is low [20].

The soluble form of RAGE should act as a decoy receptor, preventing the furthering of the pro-inflammatory cascade, in which RAGE plays a role. The role of sRAGE as a diagnostic marker for disease activity has been debated, since its levels might increase (as a way for the body itself to try to shut down inflammatory processes) in several diseases, but results have been inconclusive.

As of now, we know that RAGE plays a role in a plethora of other physiological and pathological processes, including diabetes, osteoporosis and cardiovascular diseases. Over the course of time, many different factors, including oxidative stress, were studied to explain the pathophysiology of many skin diseases characterized by chronic inflammation and frequent relapses, such as atopic dermatitis [21,22,23], psoriasis [24,25], lichen planus [26,27], vitiligo [28,29], or discoid lupus erythematosus [30]. It is well known that the clinical expression of such diseases depends on a variety of factors, both inner and outer, but, as of yet, one of these factors, namely RAGE, seems to play an important role in a context of continuous inflammatory and autoimmune stimuli, thus prompting further studies in order to complete the picture we have of such diseases.

As outlined before, there is great scientific interest and research about the importance of RAGE in all fields of medicine. Dermatology is a peculiar discipline, which includes purely cutaneous diseases and cutaneous manifestations of internal or multi-organ diseases, and dermatological research on RAGE reflects this fact, because available resources were spread over multiple studies concerning different conditions within the discipline, producing a remarkable but polyhedric amount of data. The aim of this review is to organize the currently available knowledge about the role of RAGE in the macrocosm represented by skin diseases to better understand immunopathological pathways involved and possible therapeutic options.

## 2. Methods

This review was performed by browsing the PubMed database (https://www.pubmed.gov), searching for articles labeled with both the MeSH (Medical Subject Headings) terms “RAGE” and “Skin”, with the search string *(“skin”[MeSH]) AND “Receptor for Advanced Glycation End Products”[MeSH]*. The initial search produced 39 articles, of which nine were excluded from this review by title and abstract only, and three were excluded after reading them entirely. One additional article was found and included after a search in the PubMed database with the two MeSH terms *“Vitiligo”* and *“Receptor for Advanced Glycation End Products”*, for a grand total of 28 articles. The articles included in this review showed a direct link between RAGE and various skin conditions, because RAGE was either a prominent feature in prolonging the inflammatory response or a primary cause of it. The excluded articles were reviews, did not concern a direct link between RAGE and skin, or did not provide enough information about materials and methods. Due to the great heterogeneity of materials, methods, and outcomes of the above cited studies, a meta-analysis was not deemed appropriate.

## 3. Results

**Figure 1:**
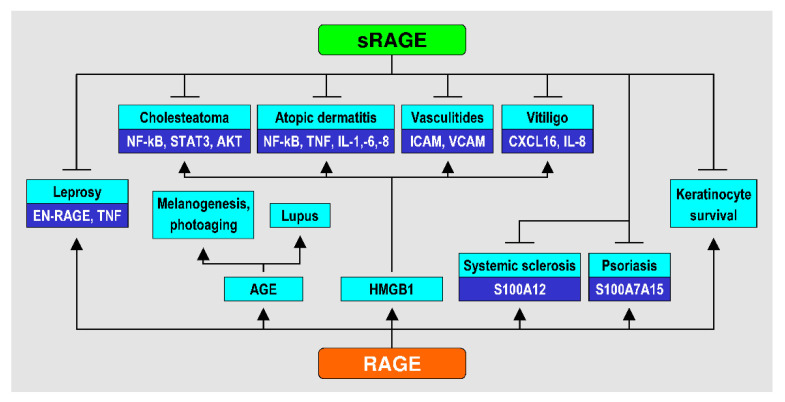
Effects of RAGE and sRAGE in various skin conditions.

A summary of the main data of the studies included in the review [20,31,32,33,34,35,36,37,38,39,40,41,42,43,44,45,46,47,48,49,50,51,52,53,54,55,56,57] is presented in Table 1. Effects of RAGE and sRAGE in various skin conditions, as reported in the said studies, are summarized in Figure 1.

### 3.1. RAGE as an Inflammation Receptor

A study by Riehl et al. [31] noted that even though RAGE is not essential for an inflammation process to start, it sustains a concerted increase of other transcriptional factors such as the Rb (retinoblastoma tumor suppressor protein)-E2F pathway, demonstrated by a more prominent staining in wild type mice than in RAGE^−/−^ ones upon stimulation with tetradecanoylphorbol acetate (TPA).

Another remarkable characteristic, demonstrated by Leibold et al. [32], is that mice with a mutation or deletion of RAGE on keratinocytes show an earlier resolution process after an initial inflammatory one, partially because of the role of RAGE in the production of TNF-α by keratinocytes. Nienhuis et al. [33] found a significant role of RAGE in lupus: sRAGE levels were significantly higher in lupus lesional skin.

Another prominent study by Wolf et al. [34] on mice demonstrated that in TPA-induced skin psoriasis lesions, proinflammatory cytokines such as MIP-2 (macrophage-inflammatory protein 2), IL-1α, and TNF-α were significantly lower if RAGE was absent. On the other hand, RAGE null mice showed a higher level of IL-1Ra (interleukin 1 receptor antagonist), as an anti-inflammatory effect, even though only in vitro.

Wolf et al. also demonstrated that in vivo administration of RAGE-blocking antibodies ameliorated the clinical score of atopic dermatitis by reducing the levels of pro-inflammatory cytokines, even though the levels of IL-1α did not drop significantly [34]. Szczepanski et al. [35] studied a very specific skin district, i.e., the ear, and the correlation between the HMGB1/RAGE pathway and the pathogenesis of cholesteatoma. Through such a chain reaction, they noted an increase of RAGE levels on skin cells, a more consistent production of IL-8, and better protection from apoptosis of keratinocytes by phosphorylation of MEK (meiotic chromosome-axis-associated kinase) 1/2, STAT3, MAPK (mitogen-activated protein kinase) p44/p22, and NF-κB. All these effects were lost when the cell medium was enriched with RAGE-binding antibodies [35]. NF-κB and the subsequent production of IL-6 were also shown to be downregulated in mice with TPA-induced atopic dermatitis, when they were treated with *Glycyrrhiza glabra*, a direct inhibitor of HMGB1, one of the main ligands of RAGE [36]. Concerning the same mechanism, it is noteworthy that de Carvalho et al. investigated the role of RAGE in lichen planus, observing that its levels were more prominent in the upper layers of the dermis of patients compared with healthy controls, even though the levels of its mRNA were lower: they suggested that this might be due to the rapid translocation of HMGB1 by affected keratinocytes [37]. On the same note, but regarding atopic dermatitis, there is a study focusing on the role of quercetin in inhibiting the RAGE pathway through HMGB1 [38]: in this study, the authors observed downregulation of cytoplasmic HMGB1, RAGE, nuclear p-NFκB, p-ERK1/2, COX2, TNFα, IL-1β, IL-2Rα, IFNγ, and IL-4 and upregulation of nuclear Nrf2, which ultimately resulted in an attenuation of the atopic dermatitis-like lesions induced by a house dust mite extract applied to the dorsal skin of NC/Nga transgenic mice. Two more studies focused, respectively, on the difference between a physiological and pathological skin conditions such as skin thickness and inflammation, finding that RAGE expression directly correlated with epidermal thickness [39], and a possible relationship with levels of oxidative stress and artery diseases, finding that skin autofluorescence is increased in stable coronary artery disease and associated with soluble RAGE [40].

### 3.2. RAGE in Infectious Diseases

One of the ligands of RAGE, namely EN-RAGE, is implicated in the inflammatory cascade involved in the pathogenesis of leprosy. The levels of both RAGE and EN-RAGE show a significant increase in the multibacillary form of leprosy, specifically in the formation of the granuloma and perpetration of the inflammatory process [41]. Another study, by Na et al., investigated the correlation between disease severity and the levels of RAGE in *Staphylococcus aureus*-induced skin lesions. In this study, the authors observed that in RAGE-deficient mice, the lesion size was significantly smaller than in wild-type mice, and the occurrence of abscesses in the skin was significantly higher in the RAGE^−/−^ population, with a consequent delay of the healing process. This interesting contrast led them to hypothesize that in a RAGE-deficient environment, there is a more rapid inflammatory response to external stimuli and lower levels of inflammatory cytokines over time. In vitro, the authors of this study observed an enhanced phagocytic activity in RAGE^−/−^ mice compared to controls, resulting in lower bacterial load at the site of infection. However, two studies appear insufficient to draw any definitive conclusions and further studies should be conducted [42].

### 3.3. RAGE in Diabetic Skin and Repairing Processes

Lohwasser et al. demonstrated that the levels of RAGE increase over time, in particular in the basal layer of the epidermis and the fibroblasts of the upper layer of the dermis (where skin regeneration occurs) of older patients more than in fetal skin, and more in sun exposed than in not exposed skin [20].

Another dermal effect on fibroblasts was demonstrated by Niu et al. [43], who studied cell turnover in diabetic wounds: the authors let dermal cells grow on both a normal and an AGE-rich extra-cellular matrix, noticing that the interaction between AGE and RAGE produced a slow-down in cellular replication. As counterproof, the authors added anti-RAGE antibodies to the culture, producing more actively replicating cells [43].

The effect of RAGE on diabetic skin is not restricted to repairing processes: the interaction of RAGE and its ligands is also supposed to have a role in microscopic vasculopathy and keratinocyte turnover, producing the typical dryness and vascular lesions over time, by accumulation of ROS [44], and the AGE–RAGE interaction may act on endothelial cells as an inhibiting stimulus for the production of prostacyclins and a positive stimulus for the production of plasminogen activator inhibitor-1, thus increasing the thrombogenic effect on larger vessels and, perhaps, on smaller ones too, through the production of reactive oxygen species [45,46,47,48,49]. These observations led some other authors to propose topical treatments and a possible role of RAGE in estimating diabetic wounds [50,51] with promising results.

### 3.4. RAGE and Systemic Sclerosis and Ulcers

There seems to also be a prominent role of the RAGE pathway in sclerotic and ulcerative processes. Yoshizaki et al. investigated the relation between levels of HMGB1 and sRAGE and disease severity in patients affected by systemic sclerosis. In detail, correlation was observed with skin fibrosis, scars, ulcers, pulmonary fibrosis, and, at a molecular level, serum levels of anti-topoisomerase 1 antibodies, IgG, CRP (C reactive protein), and ESR (erythrocyte sedimentation rate), but not TNF-α, IL-1β, and IL-6 [52], even though another study, concerning calcinotic deposits, did not produce such clear cut results [53].

Another role of RAGE as a structure-oriented receptor was demonstrated by Zhao et al. [54]: in this study, the authors delved into the role of S100A12 as a factor activated by skin dryness and its regulation during scar formation and dermal fibrosis, showing that, in fact, in case of reduced water levels it acts as a hypertrophic stimulus through the activation of RAGE. The authors used the S100A12 molecule on ear lobes in mice, showing a significant production of hypertrophic fibrous tissue through the activation of RAGE [54].

### 3.5. RAGE and Melanocytes

The RAGE induced pathway has also an important role in melanogenesis, through ligation with AGEs. The phosphorylated form of CREB, which is part of the RAGE downstream cascade, activates the CRE (cAMP response elements) consensus motifs of MITF (microphthalmia-associated transcription factor) and, consequently, tyrosinases, boosting melanin production in melanocytes [55]. On the other hand, Cui et al. demonstrated that RAGE plays an important role in the pathogenesis of vitiligo [56] and ubiquinone-induced irritation of melanocytes [57]. In vitiligo-affected patients, RAGE, out of the three PRRs (pattern recognition receptors) that bind HMGB1 (the others being the toll-like receptors TLR2 and TLR4), is the only one upregulated, and its blockade significantly reduces the levels of IL-8 and CXCL16 [chemokine (C-X-C motif) ligand 16] in keratinocytes. The authors investigated the in vitro levels of RAGE and HMGB1 after hydrogen peroxide was added to the culture: indeed, the levels of both HMGB1 and RAGE increased. Because of these results, questions arise about the possible effects of pro-oxidative therapies such as UVB phototherapy, in which there is an activation of melanocytic stem cells and down-regulation of self-directed T cells, opposed to the UV-induced, pro-inflammatory release of HMGB1 by keratinocytes [56].

## 4. Discussion

In this study, 28 articles were considered. Ten studies centered their attention on the importance of RAGE as an inflammatory stimulus, and how it is important in order to prolong the inflammatory response over time. Three of these studies focused mostly on HMGB1, in diseases such as atopic dermatitis, cholesteatoma, and lichen planus (all of them being chronic diseases).

The most notable role of RAGE is surely that of prolonging the inflammatory process. It has been demonstrated that through the RAGE-induced pathway, pro-inflammatory, and pro-survival factors are activated, such as NF-κB, and pro-inflammatory cytokines, notably TNF (tumor necrosis factor)-α and IL (interleukin)-1β, are upregulated. Beyond the pro-inflammatory effect on macrophages and monocytes, RAGE can also affect the expression of surface adhesion molecules, such as VCAM (vascular cell adhesion molecule) and ICAM (intercellular adhesion molecule), on endothelial cells.

By blocking the HMGB1 pathway using glycyrrhizin in vivo, Wang et al. found out that the accumulation of mast cells could be prevented if glycyrrhizin was injected inside the peritoneum, whilst in vitro the addition of glycyrrhizin in a medium containing mast cells activated by rmHMGB1 did prevent the Ca^2+^ influx in these cells, thus turning off the inflammatory stimulus [36]. These studies are suggesting that indeed, if such mechanism could be blocked somehow, we could turn a potential pathogenic mechanism in a treatment opportunity, e.g., by producing a reliable form of decoy receptors (namely sRAGE) to be used topically or systemically [35,36,37].

Six other studies focused mostly on cytokine production, showing how RAGE acts as a central receptor in the extension of the inflammatory response, acting as a stimulus for factors such as NF-κB, Rb-E2F, MAPK, MEK1/2, and STAT3, which act as pro-survival signals for the keratinocytes. On the other hand, in chronic inflammatory diseases, the body itself tries to shut down the disease’s own course through the production of the decoy receptor, suggesting that the impact of such conditions can be monitored or researched on a more global scale rather than being pinpointed to a specific location. Riehl suggested that, due to the complex interactions between RAGE and other pro-inflammatory factors in skin, studying this molecule just in vitro seems like a counterproductive method [31]. In fact, in the study conducted by Leibold, the authors found out that the effects of paracrine regulation could have influenced the in vitro results, even if RAGE antagonists were administered, suggesting that in order to maintain a prolonged secretion of TNF-α, cultured cells might need some other stimulus [32]. From other studies, Nienhuis noticed that sRAGE alone acts in vitro as a pro-inflammatory stimulus, whilst if paired with HMGB1 acts as a decoy receptor, preventing it from binding to RAGE [33]. This finding in lupus lesional skin suggests that, during disease activity, there are actually mechanisms trying to counteract the pro-inflammatory effect of the RAGE cascade by releasing a decoy receptor, such as sRAGE, to prevent further accumulation of AGE in the skin. Data reported by Wolf et al. [34] suggest that further data should be acquired in order to better understand the mS100a7a15 (murine S100 calcium-binding protein A7 and A15)-RAGE axis for a possible target therapy. Given the emerging importance of alarmins such as HMGB1 and both AGE and AOPP products in both the acute and chronic phase of a wide variety of skin diseases, the best solution to carry out these studies might be using live specimens that, even though more complex due to various molecule/gene interactions, prove to be a more reliable way to understand the fundamental pathological processes involving RAGE [31,32,33,34,38,39].

Finally, another study focused on the possibility of studying the behavior of sRAGE through skin autofluorescence. Since this technique can be a reliable tool to study, in a non-invasive way, the status of one’s body under an oxidative stress perspective, the authors of this study proposed that it could be used to better understand inflammatory and oxidative stress in artery diseases [40].

These studies present results that once again confirm that the modulation of the inflammatory process, especially in systemic diseases, still remains for a large part an unknown territory; one study focusing on the role of AOPP and AGE in another cutaneous disease, i.e., mastocytosis, showed that the blood levels of AGE do not increase whilst AOPP levels do. It is interesting to note though that the AOPP serum levels were higher in asymptomatic patients than in symptomatic ones, giving the possible hint that mastocytes could be inhibited through oxidative stress, or that slowly increasing levels of AOPP could prove to be as an accommodating stimulus for the host’s immune system to turn off the inflammation. However, as suggested in the study, further research must be conducted [58]. Of interest, chronic inflammatory cutaneous diseases, like psoriasis or atopic dermatitis, are associated with an increased risk of comorbidities in whose pathogenesis RAGE is involved, such as asthma [59], obesity [60], cardiovascular diseases [61], and metabolic syndrome [62]. Full details about this issue are beyond the scope of this review, but the intriguing idea that RAGE may explain, at least in part, these associations, and possibly lead to a unified pathogenetic mechanism, appears certainly worth of investigation, also for the potential therapeutic implications.

Two studies focused on the role of RAGE in infectious diseases. In leprosy, RAGE apparently predicts gravity and duration of the disease, in particular the presence of lepra reactions; however, the levels of both RAGE and EN-RAGE were not investigated after the clearance subsequent to the treatment. These observations led to the hypothesis that RAGE plays an important role in the proinflammatory response, in particular lepra reactions [41]. In the same study, the authors mentioned the in vitro toxicity towards *Brugia malayi* and compared it with the role of EN-RAGE in lepra reactions, suggesting that the supposed antimicrobial role of EN-RAGE in the case of *Mycobacterium leprae* might be due to the TNF-α released by infiltrated cells, not EN-RAGE itself. Additionally, RAGE seems to play an important role in the timing of the resolution of *Staphylococcus aureus* infections [42], meaning that both the prolongation of the inflammatory stimulus and the mitigated initial response to external pathogens seem to be affected by the presence of this specific receptor.

Many different works (eleven) investigated the effects of RAGE at a dermal and vascular level, mostly in diabetes and skin repair processes. Given the complex nature of a systemic condition such as diabetes, further studies must be conducted in order to better define the role of RAGE in such context. What we know so far is that the interaction of RAGE and AGE produces a slowdown of cell replication, mostly due to the production of reactive products of oxygen, with visible effects such as slower skin repair processes and endothelial or nerve damage [46,47]. Niu et al. used a glycosylated matrix and evaluated the effects of anti-RAGE antibodies on fibroblasts just in vitro, suggesting that samples of untreated diabetic skin should be used to study the function of the above antibodies in vivo. An interesting suggestion made in this study was to further investigate each individual’s skin in order to better understand the course of the wound healing process; however, given the intrinsic variety of AGE products, this seems a daunting task [43]. Even the polymorphisms of the RAGE encoding genes were studied, showing that some of these sequences were related to a different anti-oxidative status [48,49]. On a more positive note, topical sRAGE could be an effective treatment in diabetic wounds, leading to a faster resolution of the scarring process, even though the same authors proposed that a treatment with lentiviral vectors could lead to a more prolonged anti-inflammatory response, much better than just the topical treatment [50]. In another study, the RAGE levels in macrophages could give an estimate of wound age, possibly in combination with other inflammation-related proteins [51], thus suggesting that RAGE could have a prognostic value and be a potential target for a specific therapy.

Two other studies delved deep into the role of RAGE as a modelling-oriented receptor, particularly in scars and ulcers in systemic sclerosis [52,53]. The effect of the RAGE pathway activation in these situations seems to be the prolonged activation of fibroblasts, which induces a longer time period for scar formation and ulcer remodeling. This might happen through the production of S100A12 by keratinocytes, which causes an over-stratification of these cells, producing an abundance of tissue. The blockage of RAGE or S100A12 creates an environment similar to the wounds of the mucosal areas, moister than that found in normal skin, with slower stratification and increased skin roughness [54].

The role of RAGE in skin remodeling and systemic sclerosis is already a defined process, worth mentioning also in this review [63]: among other alarmins such as IL-33 and IL-1α, HMGB1 seems to play a major role as a ligand for the RAGE receptor. In this condition, the levels of both HMGB1 and RAGE seem to be upregulated, along with serum levels of sRAGE, playing its role as a decoy receptor (as previously described). Given the wide variety of skin lesions and scarring processes that could be prevented through adequate control of the inflammatory process, it is thanks to these observations that we can deduce that topical anti-RAGE molecules could be used also for cosmetic objectives, beyond the already cited diagnostic purpose.

Three more articles dealt with the importance of RAGE in melanocyte-related conditions, namely skin aging and discoloration and vitiligo. In the aging skin, AGEs produce an accumulation of melanocortin-1 receptor in UV-exposed areas; other than that, there is another pathway, apparently involving RAGE, in melanin production. Cells cultured in an AGE-rich medium showed an increase in factors such as MITF (microphthalmia-associated transcription factor), possibly due to the activation of CREB and ERK (extracellular signal-regulated kinases), both down the pathway of RAGE stream [55].

In vitiligo, RAGE seems to be overexpressed in the epidermal layers: its blockade with specific antibodies reduces the release of CXCL16 and IL-8, which prompt the chemotaxis of T-cells. This activation seems to be more prominent in dendritic cells in case of higher presence of HMGB1 released by keratinocytes. As dendritic cells play an important role in the activation of T-cells, HMGB1 and the binding with its receptor could lead to a cumulative effect. These observations suggest that RAGE might have a lead role also at a smaller, local level in processes where the inflammation is not set out for prolonged periods: we should look also for secondary, indirect effects with early consequences in other processes [56] such as melanogenesis, even though this field still requires a lot of research.

Finally, in another study (in vitro), Cheong et al. investigated the role of the protein S100B (S100 calcium-binding protein B) as a potential marker for melanocytic damage [57]. One of the possible receptors for the binding of extracellular S100B is indeed RAGE, but the chemical irritation of melanocytes and the subsequent release of the S100B protein did not produce remarkable RAGE-mediated effects. This finding requires further studies: even though in normal melanocytes its effects can be marginal, RAGE is present on the surface of melanoma cells and was investigated as a potential target for therapeutic purposes (inhibition of tumor growth and metastasis). Cheong et al. demonstrated a correlation between in vivo irritation and in vitro levels of S100B inside melanocytes, supporting the idea that irritation of the melanocytes can lead to the accumulation of this factor, thus promoting cell survival mechanisms [57]. Perhaps, on this final note, one can argue that the levels of S100B can be a predictive factor of the severity of conditions such as vitiligo.

## 5. Conclusions

The current knowledge about the RAGE receptor is very limited. Given the great deal of roles that this molecule plays in different types of skin diseases, what emerges from these studies is that it would be convenient to push forward the research using live models, in order to better understand the role of RAGE in a more complete and complex environment. The great deal of skin diseases which involve both AGEs and AOPPs can provide different opportunities to study the different roles played by RAGE. It is our proposition that the two most interesting diseases that can prove useful on such note would be systemic lupus erythematosus and systemic scleroderma, since both diseases have characteristic phases (acute, subacute, and chronic), which can better define the already discussed roles of AGE (mostly in chronic conditions) and AOPP (most prominent in acute processes) and their interaction with RAGE.

Another key feature emerging from these studies is that the soluble form of RAGE (sRAGE) can prove to be a potential diagnostic tool to better asses the disease activity in various skin diseases, mostly chronic, and its topical form could be used as an alternative treatment option when other topicals are not available or could provide significant side effects. Indeed, it could be interesting to perform compared studies with the current golden standard, topical therapy and sRAGE. On the other hand, based on the current knowledge about this receptor, a systemic sRAGE treatment, although fascinating, is likely to be difficult, since RAGE does not play a single role as an inflammatory receptor but as a remodeling one as well.

Finally, a potential target therapy with antibodies, anti-RAGE, as a systemic treatment, seems to be an interesting perspective for a plethora of diseases, even though further studies must be conducted. This approach could prove useful especially in those diseases where the accumulation of ROS, presence of alarmins, and overall inflammatory state could be a greater management challenge than the first pathogenetic cause, or in those acute diseases where there is a need of a preemptive stop to a self-directed harmful process (such as small vessels diseases).

## Figures and Tables

**Table 1:** Summary of methods, materials (and their sources), and conditions examined in the studies included in the review.

Reference	Methods	Material	Source	Condition
Riehl et al. [31]	PCR	skin	mice	Inflammation
Leibold et al. [32]	IHC	skin biopsies	mice	Inflammation, keratinocytes
Nienhuis et al. [33]	ELISA	skin	humans	Systemic lupus
Wolf et al. [34]	IHC	skin	mice	Psoriasis
Szczepanski et al. [35]	IHC	keratinocytes	humans, human cell lines	Cholesteatoma
Wang et al. [36]	IHC	serum	mice	Atopic dermatitis
de Carvalho et al. [37]	IHC	serum	humans	Lichen planus
Karouppagunder et al. [38]	Western blot	skin	mice	Atopic dermatitis
Iwamura et al. [39]	IHC, PCR	skin	humans	Inflammation, apoptosis
Mulder et al. [40]	ELISA	skin, serum	humans	Endothelial diseases
Kim et al. [41]	ELISA, IHC, confocal microscopy	skin	humans	Leprosy
Na et al. [42]	ELISA, FACS, histopathology	serum	mice	Staphylococcal infections
Lohwasser et al. [20]	IHC	skin	humans	Stimulation with TNF and AGE
Niu et al. [43]	Western blot	serum	humans, human cell lines	Fibroblast proliferation
Park et al. [44]	ELISA	skin, serum	mice	Repairing and aging
Yamagishi et al. [45]	PCR & nucleotides	endothelial cells	cell lines	Diabetes & vessels
Bekircan-Kurt et al. [46]	IHC	skin biopsies	humans	Diabetes & vasculitides
Bakker et al. [47]	ELISA	serum	humans	Diabetes, celiac disease
Kankova et al. [48]	PCR	skin, blood	humans	Wounds & Diabetes
Kankova et al. [49]	PCR	skin, blood	humans	Vascular dermatoses
Wear-Maggitti et al. [50]	CD31 staining	skin	mice	Wounds & Diabetes
Ji et al. [51]	IHC, Western blot	skin	mice	Diabetic wounds
Yoshizaki et al. [52]	IHC	serum	humans, mice	Systemic sclerosis
Davies et al. [53]	IHC	skin	humans	Systemic sclerosis
Zhao et al. [54]	IHC	skin	mice, human cell lines	Dermal fibrosis
Lee et al. [55]	Western blot, IF	skin, melanocytes	human cell lines	Melanogenesis
Cui et al. [56]	IHC	skin	cell lines	Vitiligo
Cheong et al. [57]	Western blot	keratinocytes, melanocytes	human cell lines	Melanocyte toxicity

IHC = immunohistochemistry, ELISA = enzyme-linked immuno sorbent assay, PCR = polymerase chain reaction, FACS = fluorescence-activated cell sorting, IF = immunofluorescence.

## Abbreviations

AGE	advanced glycosylation end-products
AOPP	advanced oxidation protein products
cAMP	cyclic adenosine monophosphate
CRE	cAMP response elements
CREB	cAMP response element-binding protein
CRP	C reactive protein
CXCL	chemokine (C-X-C motif) ligand
DAMPs	damage-associated molecular patterns
ELISA	enzyme-linked immuno sorbent assay
EN-RAGE	endogenous secretory receptor for advanced glycosylation end-products
ERK	extracellular signal-regulated kinases
ESR	erythrocyte sedimentation rate
FACS	fluorescence-activated cell sorting
HMGB1	high mobility group box 1
ICAM	intercellular adhesion molecule
IF	immunofluorescence
IHC	immunohistochemistry
IL	interleukin
IL-1Ra	interleukin 1 receptor antagonist
LPS	lypopolysaccharide
MAPK	mitogen-activated protein kinase
MEK1/2	meiotic chromosome-axis-associated kinase
MeSH	medical subject headings
MIP-2	macrophage-inflammatory protein 2
MITF	microphthalmia-associated transcription factor
mS100A7A15	murine S100 calcium-binding protein A7 (psoriasin) and A15 (koebnerisin)
NADH	reduced form of nicotinamide adenine dinucleotide
NFAT	nuclear factor of activated T-cells
NF-κB	nuclear factor kappa-light-chain-enhancer of activated B cells
PCR	polymerase chain reaction
PRRs	pattern recognition receptors
RAGE	receptor for advanced glycosylation end-products
Rb	retinoblastoma tumor suppressor protein
ROS	reactive oxygen species
S100A12	S100 calcium-binding protein A12 (calgranulin C)
S100B	S100 calcium-binding protein B
sRAGE	soluble receptor for advanced glycosylation end-products
STAT3	signal transducer and activator of transcription 3
TLR	toll-like receptor
TNF	tumor necrosis factor
TPA	tetradecanoylphorbol acetate
UV	ultraviolet
UVA	ultraviolet A
UVB	ultraviolet B
VCAM	vascular cell adhesion molecule
